# Risk factors for COVID-19 infection and disease severity in Nigeria: a case-control study

**DOI:** 10.11604/pamj.2022.41.317.34307

**Published:** 2022-04-20

**Authors:** Rowland Utulu, Ikeoluwapo Oyeneye Ajayi, Segun Bello, Muhammad Shakir Balogun, Ugochukwu Chinyem Madubueze, Idayat Temitope Adeyemi, Olajumoke Temitope Omoju, Azuka Stephen Adeke, Adetunji Olusesan Adenekan, Osarhiemen Iyare

**Affiliations:** 1 Nigeria Field Epidemiology and Laboratory Training Program, Abuja, Nigeria,; 2 Department of Community Medicine, Alex Ekwueme Federal University Teaching Hospital Abakaliki, Ebonyi State, Nigeria,; 3 Department of Epidemiology and Medical Statistics, Faculty of Public Health, College of Medicine, University of Ibadan, Ibadan, Nigeria,; 4 Epidemiology and Biostatistics Research Unit, Institute of Advanced Medical Research and Training, College of Medicine, University of Ibadan, Ibadan, Nigeria,; 5 African Field Epidemiology Network, Abuja, Nigeria,; 6 Department of Ophthalmology, College of Medicine, University of Lagos, Lagos, Nigeria

**Keywords:** Risk factors, COVID-19, SARS-CoV-2, case-control studies, Nigeria

## Abstract

**Introduction:**

identifying risk factors for SARS-CoV-2 infection and disease severity is critical to developing measures to protect vulnerable groups. We aimed to identify risk factors for SARS-CoV-2 infection and severe disease.

**Methods:**

this was an unmatched case-control study that recruited participants in the country from April to July 2020. Cases tested positive on Reverse-Transcription Polymerase Chain Reaction (RT-PCR), while controls tested negative on RT-PCR. Data were collected by trained research assistants using an interviewer-administered questionnaire. Cases were categorized into severe and non-severe to identify risk factors for severe disease.

**Results:**

there were 497 cases and 997 controls recruited. Contact with a symptomatic confirmed case adjusted odds ratio (aOR) 1.91 (95% CI: 1.30-2.80) and attendance of mass gatherings aOR 1.74 (95% CI: 1.10-2.74) were associated with SARS-CoV-2 infection, while the use of private transportation aOR 0.56 (95% CI: 0.37-0.85) and use of a face mask aOR 0.43 (95% CI: 0.19-0.95) were associated with lower odds of infection. We identified 38 (7.7%) severe cases and 459 (92.3%) non-severe cases. Multivariate analysis identified age ≥ 50 years aOR 4.54 (95% CI: 1.86-11.08), male sex aOR 2.95 (95% CI: 1.07-8.11), hypertension aOR 3.52 (95% CI: 1.46-8.50), and diabetes aOR 5.76 (95% CI: 2.01-16.50) as risk factors for severe disease, while Hausa ethnicity aOR 0.15 (95% CI: 0.04-0.62) lowered the odds of severe disease.

**Conclusion:**

our findings highlight the importance of exposure history, mass gatherings, private transportation, and the use of face masks. Being over 50 years, male and having comorbidities indicate a worse prognosis.

## Introduction

The COVID-19 pandemic has been one of the most significant events of the 21^st^ century. As of 28^th^ January 2022, there were a total of 364,191,494 confirmed cases of COVID-19, including 5,631,457 deaths reported to the World Health Organization (WHO) worldwide [1]. In Nigeria, a total of 252,753 confirmed cases of COVID-19 and 3,134 deaths have been reported [1]. Although it is generally believed that anyone can contract the disease, studies have identified risk factors for SARS-CoV-2 infection to be male gender [2-5], age over 60 and 70 years [2,3,5], living in urban areas [2-4] and contact with a confirmed case of COVID-19 [2,3,6]. Research has shown advanced age, male sex, comorbidities such as hypertension, diabetes, obesity, cancer, and chronic kidney to be risk factors for severe COVID-19 illness [3,7,8].

The early phase of the COVID-19 pandemic was marked by widespread fear and panic occasioned by the rapid spread of the virus and the deaths that occurred. Countries lacked adequate knowledge on prevention and control strategies that were effective against this emerging disease. Resource-poor countries aiming to contain the spread of the virus relied on public health recommendations from the WHO and more advanced countries. Some States in Nigeria went into a lockdown early in the first half of the year 2020 [9], despite varied opinions on the eventual impact of the lockdown on transmission rates. One fact that soon became evident was the huge negative impact the lockdown had on the economy [9,10]. Despite public health recommendations to the contrary, Nigeria eased its lockdown in May 2020, largely on account of the severe negative impact on the economy [9,10]. Consequently, several public health measures recommended limit transmission included social and physical distancing, avoidance of mass gatherings, and use of face masks in public places. However, there is still a paucity of local evidence supporting these recommendations. The benefit of such evidence can provide policy support as well as improve adherence of the public to safe practices that may reduce transmission. The results from such studies can also provide evidence to develop targeted interventions to limit mortality from COVID-19. Although studies have identified risk factors for severe disease and mortality, few have been conducted in Nigeria. Most of the studies conducted have been subnational studies, with obvious questions regarding generalizability. We, therefore, aimed to identify risk factors for COVID-19 infection and disease severity in Nigeria.

## Methods

**Figure 1:**
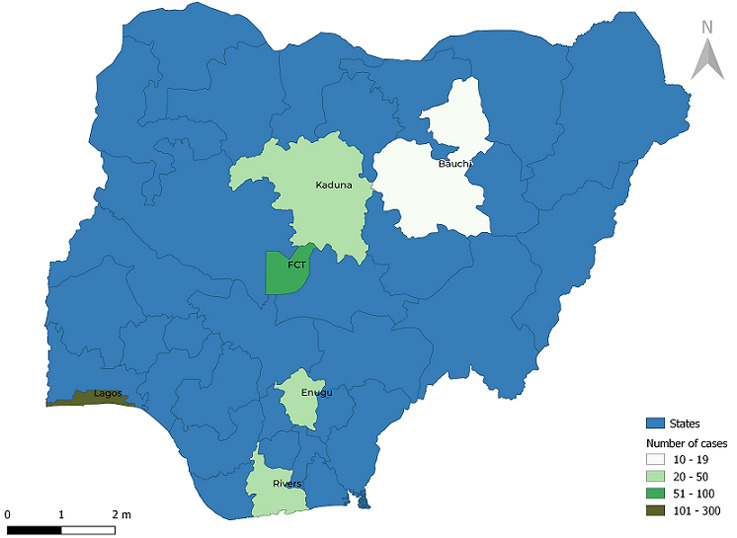
map of Nigeria showing the distribution of sampled cases from six States

**Study design, definitions, and participants:** in this 1: 2 unmatched case-control study, we recruited confirmed cases that were individuals who tested positive for SARS-CoV-2 on Reverse-Transcription Polymerase Chain Reaction (RT-PCR) from April to July 2020. Controls were contacts of confirmed cases that tested negative on RT-PCR from April to July 2020. We excluded all individuals with at least one inconclusive test result to avoid misclassification bias. The use of participants tested from April to July 2020 was aimed at including the widest variety of cases and controls, as these were some of the first person exposed and confirmed to have COVID-19 in the country. This improved the probability of recruiting imported cases, those infected following contact with imported cases and the resultant community transmission. In the absence of access to patient health records, severe cases were classified as those who had self-reported difficulty breathing, required respiratory support, ICU admission, or died from complications of the disease [11]. Non-severe cases were those who were asymptomatic, and had symptoms other than difficulty in breathing. The minimum sample size was calculated using the formula for a case-control study with unequal sizes. We calculated the minimum sample size for each associated factor for COVID-19 infection and disease severity that had been previously stated. Hypertension was selected as the variable with the largest estimated sample size of 451, using the proportion of COVID-19 cases with hypertension of 0.36 [4], 5% type 1 error rate, 80% power, and 10% non-response rate. Therefore, the total sample size required for cases was 496 and 992 controls. Multistage sampling technique was used to select participants. In the first stage, the country was stratified into six geopolitical zones, and one state was selected per geopolitical zone using simple random sampling by balloting. Lagos, Rivers, Abuja, Kaduna, Enugu, and Bauchi States were selected. In the second stage, proportionate sampling was first done to determine the number of participants to be selected per state, based on their proportionate contribution in the population of confirmed cases, and then simple random sampling using computer-generated random numbers was used to select the participants. The distribution of cases sampled in each of the six states is depicted in Figure 1.

**Data collection:** persons who met the Nigeria Center for Disease Control (NCDC) suspect case definition were tested for SARS-CoV-2 using RT-PCR. Sociodemographic data and relevant exposure history were collected from each suspect by field epidemiologists. Then nasopharyngeal or nasal swabs were collected for that suspect case, stored at 2-4°C, and sent to the laboratory using triple-packaging under aseptic conditions. Anonymized data on the participants was provided by the Nigeria Center for Disease Control (NCDC) from its online database, Surveillance Outbreak Response Management and Analysis System (SORMAS) following administrative and ethical approval. This database contains the sociodemographic, clinical history, as well exposure history of all persons tested for SARS-CoV-2. Data was collected over the phone from November to December 2020 by trained clinical medical students using an adapted electronic semi-structured interviewer-administered questionnaire. To address recall bias, the information obtained from each participant was compared with that provided on SORMAS where available. When there was disparity, the information provided on SORMAS was used, as it was believed to be less prone to issues of recall.

**Data analysis:** data were extracted from the online server in a Microsoft Excel spreadsheet before analysis, cleaned, and then read into statistical package for social sciences (SPSS) version 26. Mean and standard deviation (SD) were used to summarize normally distributed continuous variables, while median and interquartile range (IQR) were used where continuous variables were not normally distributed. Categorical variables were summarized using frequencies and proportions. The differences in proportions between cases and controls were tested using Chi-square where appropriate, otherwise Fisher´s exact was used, while the Student t-test was used for continuous variables. P-values of 0.05 or less were considered statistically significant. The magnitude of association between variables was represented using the odds ratio and their respective 95% confidence intervals. All variables included in the final multivariate model had to satisfy biologic plausibility, have a variance inflation factor of less than 3.0, and P-values of 0.2 or less.

**Ethic approval:** phone calls were made to participants explaining the study aims, benefits, risks, and voluntaries of participation. Written informed consent was requested for each participant who accepted, and was only waived upon request by participants who preferred to provide verbal consent. Assent was obtained from all participants less than 18 years where it could be provided, and informed consent was obtained from their parents. Ethical approval for the study was approved by the Institutional Review Board of the Nigeria Institute of Medical Research with the project number IRB/20/079.

## Results

**Sociodemographic characteristics of cases and controls:** we recruited 1494 participants; 497 cases and 997 controls. Most of the cases 407 (81.9%) and controls 832 (83.5%) were less than 50 years of age. There were more male cases 310 (62.4%) than controls 555 (55.7%), p= 0.013, and those who had ever been married predominated among both cases 339 (68.2%) and controls 682 (68.4%). Majority of both cases 396 (79.7%) and controls 794 (79.6%) had at least a tertiary level of education and were Christians, with 362 (75.9%) cases vs 759 (76.8%) controls. Yoruba ethnic group constituted 190 (38.2%) cases vs 390 (39.1%) controls, p= 0.001. Although health workers constituted a small proportion of all occupations, there were proportionately more among cases 102 (20.5%) than controls 183(18.4%) (Table 1).

**Table 1:** sociodemographic characteristics of cases and controls

Variables	Case N= 497 n (%)	Control N= 997 n (%)	X^2^	P-value
Mean age ± SD (years)	37.7 ± 11.7	38.1± 11.7		0.2309
**Age group (years)**				
Age < 50	407 (81.9)	832 (83.5)		
Age ≥ 50	90 (18.1)	165 (16.5)	0.482	0.487
**Sex**				
Male	310 (62.4)	555 (55.7)	6.121	0.013*
Female	187 (37.6)	442 (44.3)		
**Ethnicity**				
Igbo	101 (20.3)	268 (26.9)	16.531	0.001*
Yoruba	190 (38.2)	390 (39.1)		
Hausa	59 (11.9)	130 (13.0)		
Others	147 (29.6)	209 (21.0)		
**Marital status**				
Ever married	339 (68.2)	682 (68.4)	0.006	0.939
Single	158 (31.8)	315 (31.6)		
**Religion**				
Islam	115 (25.1)	229 (23.2)	0.155	0.694
Christianity	362 (75.9)	759 (76.8)		
**Educational status**				
Secondary and lower	101 (20.3)	203 (20.4)	0.986	0.251
Tertiary	396 (79.7)	794 (79.6)		
**Occupation**				
Health workers	102 (20.5)	183 (18.4)	1.010	0.315
Others	395 (79.5)	814 (81.6)		

* Significant variables

**Clinical characteristics and exposure history of cases and controls:** most cases were non-smokers 472 (95%) as were the controls 955 (95.8%). One hundred and twenty-three (24.7%) cases had a pre-existing disease condition compared to 163 (16.3%) controls and this difference was significant p = <0.0005. Hypertension and diabetes were the most prevalent pre-existing medical conditions, with 62 (12.5%) hypertensive cases vs 101 (10.1%) hypertensive controls and 27 (22%) diabetic cases vs 31 (3.1%) diabetic controls respectively, with the difference in the composition of diabetics among cases and controls being statistically significant p= 0.029. Proportionately more cases 256 (51.5%) compared to controls 145 (14.5%) had symptoms, p <0.0005. Among those who had symptoms, fever was the most prevalent symptom among cases 143 (55.9%) and controls 73 (50.3%), p<0.0005. The use of face masks by respondents in this study was high with proportionately fewer cases 467 (94%) using face masks compared to controls 963 (96.6%), and this difference was statistically significant p = 0.018. A lower proportion of cases than controls reported contact with a confirmed case, 168 (33.8%) cases vs 474 (47.5%) controls, p <0.0005. However, proportionately more cases 105 (63.6%) reported exposure to a symptomatic confirmed case than controls 224 (47.4%), p <0.0005, as well as more cases 59 (18%) reported exposure to a person with respiratory illness who was never tested compared to controls 67 (12.8%), p= 0.01. There were statistically significant differences in other exposures such as contact with animals among cases 62 (12.5%) vs controls 168 (16.9%), p=0.027, eating partially cooked meat (“Suya”) with cases 104 (20.9%) vs controls 146 (14.6%), p=0.002, and the use of private transportation with cases 303 (62.3%) compared to controls 753 (75.5%), p <0.0005. No significant differences were observed between cases and controls in regard to the history of travel to COVID-19 endemic countries, attendance of mass gatherings outside the country of residence and at mass gatherings within the country of residence, or with the use of public transportation (Table 2).

**Table 2:** clinical characteristics and exposure history of cases and controls

Variables	Case N= 497 n (%)	Controls N=997 n (%)	X^2^	P-value
**Smoking history**				
Current smoker	25 (5)	42 (4.2)	0.518	0.472
Non-smoker	472 (95)	955 (95.8)		
**Pre-existing disease**	123 (24.7)	163 (16.3)	15.117	**<**0.0005*
Hypertension	62 (12.5)	101 (10.1)	1.876	0.17
Diabetes	27 (5.4)	31 (3.1)	4.798	0.029*
Symptoms present before illness or test for COVID-19	256 (51.5)	145 (14.5)	230.796	<0.0005*
**Type of symptoms present**	**N=256 (51.5)**	**N=145 (14.5)**		
Fever	143 (55.9)	73 (50.3)	123.395	<0.0005*
Cough	106 (41.4)	32 (22.1)	129.867	<0.0005*
Fatigue	102 (39.8)	25 (17.2)	138.397	<0.0005*
Sore throat	52 (20.3)	16 (11)	59.902	<0.0005*
Chest pain	51 (19.9)	10 (6.9)	72.596	<0.0005*
Anosmia	92 (35.9)	38 (3.8)	90.211	<0.0005*
Ageusia	67 (26.2)	33 (26.2)	54.937	<0.0005*
Difficulty breathing	63 (24.6)	9 (6.3)	100.224	<0.0005*
Headache	99 (38.7)	42 (29)	95.734	<0.0005*
Diarrhoea	15 (5.9)	4 (2.8)	18.09	<0.0005*
Vomiting	9 (3.5)	1 (0.6)	14.596	<0.0005*
Contact with a person with respiratory illness	59 (11.9)	67 (6.7)	11.396	0.01*
Contact with a confirmed case	168 (33.8)	474 (47.5)	25.550	<0.0005*
Contact with a symptomatic confirmed case	105 (63.6)	224 (47.4)	12.980	<0.0005*
Contact with any animal	62 (12.5)	168 (16.9)	4.876	0.027*
Eat raw or partially cooked meat (''Suya'')	104 (20.9)	146 (14.6)	9.393	0.002*
Travel outside country of residence	18 (3.6)	35 (3.5)	0.012	0.913
Attended mass gatherings outside country	21 (4.2)	29 (2.9)	1.777	0.18
Attended mass gathering within your country	126 (25.4)	218 (21.9)	2.275	0.13
Private transportation	303 (62.3)	753 (75.5)	27.687	**<**0.0005*
Public transportation	227 (46.7)	498 (49.9)	1.374	0.24
Use of face mask	467 (94.0)	963 (96.6)	5.578	0.018*

* Significant variables

**Risk factors for SARS-CoV-2 Infection:** in the adjusted model, odds ratio for SARS-CoV-2 infection was higher among persons who attended mass gatherings within the country (aOR = 1.74; 95% CI= 1.10-2.74), and among those who had contact with a symptomatic confirmed case (aOR = 1.91; 95% CI= 1.30-2.80). However, use of private transportation (aOR = 0.56; 95% CI= 0.37-0.85) and use of a face mask (aOR = 0.43; 95% CI= 0.19-0.95) were both associated with lower odds of SARS-CoV-2 infection (Table 3).

**Table 3:** risk factors for SARS-CoV-2 infection

Variables	Crude OR (95% CI)	Adjusted OR (95% CI)
**Sex**		
Male	1.32 (1.06, 1.65)	1.46 (0.99, 2.15)
Female	1	1
**Ethnicity**		
Igbo	1.87 (1.37, 2.55)	1.28 (0.74, 2.21)
Yoruba	1.44 (1.01, 1.90)	1.24 (0.75, 2.05)
Hausa	1.55 (1.07, 2.25)	1.29 (0.70, 2.37)
Other ethnicity	1	1
Hypertension	1.26 (0.90, 1.77)	0.78 (0.43, 1.39)
Diabetes	1.79 (1.06, 3.03)	1.62 (0.67, 3.88)
Attended mass gathering outside country	1.47 (0.83, 2.61)	0.74 (0.21, 2.65)
Attended mass gathering within country	1.21 (0.94, 2.56)	1.74 (1.10, 2.74)*
Contact a symptomatic confirmed case	1.95 (1.35, 2.80)	1.91 (1.30, 2.80)*
Contact with an animal	0.70 (0.51, 0.96)	0.68 (0.41, 1.10)
Ate raw or partially cooked meat (''Suya'')	1.54 (1.17-2.04)	1.28 (0.81, 2.05)
Use of private transportation	0.54 (0.43, 0.68)	0.56 (0.37, 0.85)*
Use of face mask	0.55 (0.33, 0.91)	0.43 (0.19, 0.95)*

* Significant variables

**Demographic and baseline characteristics of confirmed cases by disease severity:** there were 38 (7.6%) severe and 459 (92.4%) non-severe cases. There were significant differences in the composition of severe and non-severe cases in those aged over 50 years 25 (65.8%) vs 65 (14.2%), p<0.0005, who were males 31 (81.6%) vs 279 (60.8%), p =0.011, had ever been married 36 (94.7%) vs 303 (66.0%), p <0.0005 and Muslim 16 (42.1%) vs 99 (22.6%), p= 0.007 respectively. There were also significant differences in the proportion of severe and non-severe cases among those with pre-existing disease 28 (73.7%) vs 95 (20.7%), p <0.0005, hypertension 18 (47.4%) vs 44 (9.6%), p <0.0005 and diabetes 12 (31.6%) vs 15 (3.3%), p <0.0005 respectively. There were no significant differences in the severe and non-severe cases by educational and occupational status (Table 4).

**Table 4:** sociodemographic and baseline characteristics of confirmed cases by disease severity

Variables	Severe cases N=38 n (%)	Non-severe cases N= 459 n (%)	X^2^	P-value
**Age group**				
Age < 50	13 (34.2)	394 (85.8)		
Age ≥ 50	25 (65.8)	65 (14.2)	63.08	<0.0005*
**Sex**				
Male	31 (81.6)	279 (60.8)	6.47	0.011*
Female	7 (18.4)	180 (39.2)		
**Ethnicity**				
Igbo	6 (15.8)	95 (20.7)	19.72	<0.0005*
Yoruba	10 (26.3)	180 (39.2)		
Hausa	13 (34.2)	46 (10.0)		
Others	9 (23.7)	148 (30.1)		
**Marital status**				
Ever married	36 (94.7)	303 (66.0)	13.35	<0.0005*
Single	2 (5.3)	156 (34.0)		
**Religion**				
Islam	16 (42.1)	99 (22.6)	7.31	0.007*
Christianity	22 (57.9)	340 (77.4)		
**Educational status**				
Secondary or lower	9 (23.7)	99 (20.0)	0.29	0.59
Tertiary	29 (76.3)	367 (80.0)		
**Occupational status**				
Health worker	6 (15.8)	96 (20.9)	0.57	0.45
Others	32 (84.2)	363 (79.1)		
**Pre-existing disease**	28 (73.7)	95 (20.7)	52.90	<0.0005*
**Hypertension**	18 (47.4)	44 (9.6)	45.88	<0.0005*
**Diabetes**	12 (31.6)	15 (3.3)	54.75	<0.0005*

* Significant variables

**Risk factors for severe SARS-CoV-2 disease:** adjusted odds ratio for severe COVID-19 disease was higher among persons over the age of 50 years (aOR = 4.54; 95% CI= 1.86-11.08), those who were males (aOR= 2.95; 95% CI= 1.07-8.11), hypertensive (aOR = 3.52; 95% CI= 1.46-8.50) and diabetic (aOR= 5.76; 95% CI= 2.01-16.50). Adjusted odds ratio for severe outcomes was lower among those of Hausa ethnicity (aOR= 0.15; 95% CI= 0.04-0.62). The odds of severe outcome did not differ significantly for marital status or religion (Table 5).

**Table 5:** risk factors for severe SARS-CoV-2 disease

Variables	Crude OR (95% CI)	Adjusted OR (95% CI)
**Age group (years)**		
Age ≥ 50	11.66 (5.68, 23.94)	4.54 (1.86, 11.08)*
Age < 50	1	1
**Sex**		
Male	2.86 (1.23, 6.63)	2.95 (1.07, 8.11)*
Female		1
**Ethnicity**		
Igbo	1.35 (0.45, 4.04)	1.01 (0.29, 3.53)
Yoruba	1.25 (0.48, 3.25)	1.32 (0.44, 3.96)
Hausa	0.24 (0.09, 0.65)	0.15 (0.04, 0.62)*
Other ethnicity	1	1
**Marital status**		
Ever married	9.27 (2.20, 38.99)	2.38 (0.51, 11.27)
Single	1	1
**Religion**		
Islam	2.50 (1.26, 4.94)	0.61 (0.19, 1.97)
Christianity	1	1
Hypertension	8.49 (4.18, 17.24)	3.52 (1.46, 8.50)*
Diabetes	13.66 (5.81, 32.15)	5.76 (2.01, 16.50)*

* Significant variables

## Discussion

In this analysis of 497 cases and 997 controls recruited from six States within the country, risk factors for SARS-CoV-2 infection were contact with a symptomatic confirmed case and attending mass gatherings within the country, while the use of a face mask and private transportation were associated with reduced odds of infection. Our sub-analysis of cases in this study identified age of 50 years or greater, male sex, hypertension, and diabetes as risk factors of severe SARS-CoV-2 disease while being of Hausa ethnicity was found to be associated with lower odds of severe disease. Previous studies found exposure or contact with a confirmed case to be a risk factor for COVID-19 infection [12-14]. For lack of a more complete database of individuals who tested negative, contacts of confirmed cases were used as controls in this study, and this prevented the usual assessment of exposure to confirmed cases as a risk factor for the infection. This was because contacts were selected as controls on account of their negative RT-PCR test despite exposure, while some cases were unsure or could not remember if they were ever exposed, which led to lowered odds of infection. Consequently, contact with a symptomatic confirmed case among cases and controls was assessed and this was positively associated with the infection. This further contributes to the evidence on the importance of symptoms in the transmission of COVID-19. Despite studies demonstrating the possibility of asymptomatic transmission, our study showed the greater importance of symptoms in transmission [15,16]. This provides evidence for the use of symptoms for screening in public places and occupational settings. Discharging infectious materials into the immediate environment could be enhanced in the presence of symptoms such as sneezing and coughing. The World Health Organization defined mass gatherings as “Events characterized by the concentration of people at a specific location for a specific purpose over a set period of time that have the potential to strain the planning and response resources of the host country or community” [17]. The findings of this study are similar to those of previous studies in Europe, Asia, and Malawi that found attending a mass gathering, or public and social events to increase the odds of SARS-CoV-2 infection [18-20]. While attending a mass gathering within the country was shown to be a risk factor for SARS-CoV-2 infection, attending a mass gathering outside one´s country was not associated with the infection. This may have been the result of stricter guidelines and enforcement of public health and safety measures such as handwashing and compulsory use of face masks in public places in western countries. The pandemic began later in Nigeria than in Asia, Europe, and the US, with gradual enforcement of public health and safety measures. Despite the restriction of movement and lockdown that ensued, private gathering, parties, and some social events continued, albeit with smaller numbers of persons present. This may have led to increased transmission of infection among persons attending such events in the country. After the easing of the lockdown in major States in the country, the government published recommendations regarding social distancing, use of face masks, compulsory handwashing, and use of hand sanitizers before entering public facilities and regulation of the numbers of persons in public places such as churches and banks. These were met with groans from the public, particularly the clergy. Our findings show that mass gatherings contributed to the spread of the virus in Nigeria. Therefore, measures previously instituted to safeguard public health such as physical distancing and face masks may still prove valuable especially given widespread vaccine hesitancy. Public transportation systems which mainly include cars and buses in our environment are often crowded and this increases person-to-person contact, with a greater risk of transmission [21,22]. Our study demonstrated a 48% reduction in risk of contracting SARS-CoV-2 among those who used private transportation. Although it is generally understood that public transportation can predispose to various respiratory diseases, few actual studies except modelling studies have shown increased risk or reduction in risk when using private transportation [12,22]. For most resource-poor countries public transportation is the main source of transportation, and only a few can afford private transportation. Consequently, public health measures such as physical distancing, the use of face masks, handwashing, and the use of hand sanitizers that could reduce the risk of transmission in public transportation are strongly recommended.

The reported use of face masks in this study was high with over 90% of both cases and controls reporting use. This use of face masks in our study was associated with a 57% reduction in odds of SARS-CoV-2 infection, which was similar to findings from other studies around the world [23-25]. Face mask remains one of the most important preventive measures against the infection, especially when social gatherings cannot be avoided. This underlines the recommendations of public health experts to continue the use of face masks despite the discovery of potent vaccines against SARS-CoV-2.

Persons aged 50 years or more were identified in our study to have significantly higher odds of severe COVID-19 disease. This is similar to the finding from several other studies that identified advancing age to be significantly associated with adverse outcomes [8,26-28]. The main reason for the higher odds of severe disease stated in literature is the decrease in immunity with advancement in age [29,30]. This decrease in immunity has also been associated with angiotensin-converting enzyme 2 (ACE-2) which is thought to confer protection against lung infections but is reduced in older adults [30]. Some studies suggested the confounding role of comorbidities on the relationship between age and severe SARS-CoV-2 disease, which was shown by other studies to persist after controlling for comorbidities, as was the case in this study [31,32].

Similar to previous studies around the world, male sex was identified to be an independent risk factor for severe SARS-CoV-2 disease [5,8,30,33]. Scientists have opined different reasons for this finding such as differences in sex-mediated immune response with reduced resistance to bacterial and viral infections in males and differential expression of ACE2 between the sexes [8,33].

One source stated that the testicles in males serve as a sanctuary site for SARS-CoV-2 and delay the clearance of the organism [34]. Another article cited the higher levels of inflammatory markers associated with myocardial and kidney injury such as LDH, CRP and fibrinogen in males compared to females [35]. Among those infected with SARS-CoV-2, people with diabetes had the greatest odds of adverse outcomes with 5.76-fold higher odds. Confirmed cases with hypertension also had 3.52-fold higher odds of severe disease. These findings are comparable to previous studies that reported greater odds of adverse outcomes among people with comorbidities like hypertension and diabetes [5,6]. These metabolic diseases are associated with impairment in innate immunity. This immune impairment is associated with CD8+ T lymphocyte dysfunction and a cytokine storm [36]. This results in persistent inflammation with damage to the respiratory system, among others [36,37]. Some other immune changes seen with hypertension and diabetes include deregulation of ACE2 inhibitors which function to limit inflammation [37,38]. In the absence of ACE2 inhibitors, there is unchecked systemic inflammation due to the expression of ACE2 with increased binding sites for SARS-CoV-2 [37,38]. Evidence on risk factors for severe disease is useful in the identification of vulnerable groups for vaccination, as well as prioritized clinical care. During the first wave of the COVID-19 pandemic in Nigeria, isolation centers were frequently overrun, because they were used to isolating positive persons regardless of disease severity. Consequently, some vulnerable groups such as those above 50 years of age or more and those with comorbidities were occasionally unable to access institutionalised care. These vulnerable groups should be managed in health facilities rather than home care due to the tendency towards adverse outcomes. It was unclear why Hausa ethnicity was associated with a lower odd of severe disease as the median age, the prevalence of comorbidities was comparable to other ethnic groups.

**Limitations:** the criteria used to assess SARS-CoV-2 severity without the recourse to patient health records, radiologic and laboratory data makes comparison with some other studies difficult. However, the results of our study mirror other studies that used more exhaustive criteria for assessing the disease severity. The retrospective nature of this study may have made it prone to recall bias. We tried to limit recall bias by relying on information available on SORMAS, when they were discrepancies in the information provided during data collection, and at the presentation when samples were collected.

**Funding:** funding for data collection and development of the manuscript was jointly provided by AFENET and the authors.

## Conclusion

We found that those who had contact with a symptomatic confirmed case or attended a mass gathering were more likely to have SARS-CoV-2 infection than those who did not. Conversely, people who used private transportation and wore face masks were less likely to get infected. Also, people aged 50 and more were more likely to have a severe COVID-19 than younger people. Male, hypertensive or diabetic persons were also more likely to have a severe illness. However, people of Hausa ethnicity seemed to be less likely to have a severe illness compared to people of other ethnicities. These findings underline public health recommendations of public mask-wearing, social and physical distancing, particularly for persons over 50 years of age and with comorbidities, as well as prioritizing the use of private transportation. The recruitment of participants from one state in each geopolitical zone of the country and those tested between April to July 2020 are likely to improve the generalizability of our study.

### What is known about this topic

- Risk factors for SARS-CoV-2 infection in advanced economies;
- Risk factors for SARS-CoV-2 disease severity in advanced economies;
- Use of face mask lowers the risk of infection.

### What this study adds

- This study highlights the importance of symptomatic transmission of SARS-CoV-2;
- The study also highlights the importance of private transportation system in decreasing the risk of infection with SARS-CoV-2;
- Our study found Diabetes to be the most significant risk factor for severe disease.
